# An exploratory data analysis method to reveal modular latent structures in high-throughput data

**DOI:** 10.1186/1471-2105-11-440

**Published:** 2010-08-27

**Authors:** Tianwei Yu

**Affiliations:** 1 Department of Biostatistics and Bioinformatics, Rollins School of Public Health, Emory University, Atlanta, USA

## Abstract

**Background:**

Modular structures are ubiquitous across various types of biological networks. The study of network modularity can help reveal regulatory mechanisms in systems biology, evolutionary biology and developmental biology. Identifying putative modular latent structures from high-throughput data using exploratory analysis can help better interpret the data and generate new hypotheses. Unsupervised learning methods designed for global dimension reduction or clustering fall short of identifying modules with factors acting in linear combinations.

**Results:**

We present an exploratory data analysis method named MLSA (Modular Latent Structure Analysis) to estimate modular latent structures, which can find co-regulative modules that involve non-coexpressive genes.

**Conclusions:**

Through simulations and real-data analyses, we show that the method can recover modular latent structures effectively. In addition, the method also performed very well on data generated from sparse global latent factor models. The R code is available at http://userwww.service.emory.edu/~tyu8/MLSA/.

## Background

Modularity refers to the organization of biological units (genes, proteins etc.) into quasi-autonomous groups [1]. It is an abstract concept that may take different forms in different networks. In systems biology, the most common modular structures are co-regulated genes by common transcription factors (TFs) [2-4], proteins that interact with common hub proteins [5,6], and metabolites in the same metabolic pathway [7]. Unsupervised learning methods, such as methods for dimension reduction and clustering, are used to find underlying data structures [8,9], and generate lower-dimensional data for downstream analysis [10-12]. Given the modular organization of the network, the ideal structure estimation and dimension reduction should capture local signals, rather than vague global signals that do not reflect the true properties of the network.

To understand the modules, the key is to find the activity levels of the controlling nodes. However the activity levels, e.g. transcription factor (TF) activities in gene expression, are not directly measured. Studies that incorporate TF-gene linkage databases with gene expression data showed that multiple TFs can act on a gene, and the expressions of the genes within a module regulated by the same set of TFs can be modeled reasonably well by linear functions with proper data transformation [13,14]. These studies also suggested that the transcription levels of the TFs themselves generally do not reflect the activity levels, which argues for the usage of latent variable models. Given the high dimensionality of the data and the high noise level, the success of such models relies on the availability of prior knowledge about the network topology. However, the knowledge in TF-gene relationships is still scarce for many organisms. In addition, for measurements taken at the protein or metabolite level, it is hard to define such causal linkages, as the controlling factors are not easy to pinpoint. Hence we ask the question: given a matrix of expression levels alone, can we identify hidden factors that work in combinations to exert control over subgroups of biological units? The loading matrix of a modular system should be sparse, because the modular organization confines the impact of most of the controlling factors to be local rather than global. In addition, the non-zero loadings should form blocks, with every block corresponding to one module.

Methods for the identification of tight clusters, such as gene-shaving [15], bi-clustering [16] and context-dependent clustering [17], cannot identify hidden factors that act in linear combinations. The factor model framework allows linear combinations of factors to act on each gene. Traditional methods in this area, such as principal component analysis (PCA), independent component analysis (ICA), Bayesian decomposition [18] etc, are of limited use because they do not enforce sparsity on the loading matrix. Loading matrix sparsity can be achieved through penalization in sparse principal component analysis (SPCA) [19], and proper sparsity priors in sparse Bayesian factor models [20]. However these methods do not enforce block structures in the loading matrix. Here we describe a projection-based method for the identification of modular latent structures. We refer to the method as MLSA (Modular Latent Structure Analysis) in this manuscript.

## Methods

The goal of our method is to find a collection of low-dimensional subspaces that explain the expression of subgroups of genes very well. Consider a data matrix ***G**_p × n _with p *genes measured at *n *conditions. Our goal is to find a series of orthonormal basis $\left\{B_{n\times k_{j}}^{\left(j\right)}\right\}$, where *j *is the index of the basis, and *k_j _*is the dimensionality of the *j^th ^*basis, such that with each ***B ***matrix, a subgroup of the genes have large proportions of their variation explained by the subspace defined by ***B***. We first describe the objective function and the corresponding optimization method for the identification of a single module with known or assumed dimensionality. We then describe a forward-selection scheme to identify a module when the dimensionality is unknown. In addition, an overall workflow for finding multiple modules in a dataset is presented.

The MLSA method requires that all expression vectors are standardized to have length 1. The exact standardization depends on the data properties and assumptions. The easiest is to simply scale each row vector of the matrix to achieve length 1. Column-wise normalization such as mean removal or quantile normalization could be performed in order to remove large experimental bias, and row-wise mean removal could be performed if the user considers only relative changes in each gene is important. After standardization, when seeking a subspace ***B***, the length of the projected vector in the subspace can be used to judge the amount of variation explained by the subspace.

We use ***g**_i _*to denote the expression vector of the *i^th ^*gene, and *l_i _*to denote its projection length. Given ***B ***= (***β***_1_,***β***_2_,...,***β***_k_), where the ***β'*s **are unit vectors orthogonal to each other, and *k *is the number of dimensions of the subspace,

(1) li=(gi'β1)2+(gi'β2)2+...+(gi'βk)2

### The objective function

In the search for a matrix ***B***, the true module membership information is missing. Ideally genes not belonging to the module should not contribute to the estimation of ***B***. Thus the problem is estimation in the presence of a latent variable (module membership). To address this issue, we adopt the intuition of the expectation-maximization (EM) algorithm [21], although no explicit likelihood function is assumed. A weight is defined as a function of the projection length, *w_i _*= *h*(*l_i_*), to reflect the belief of whether a gene belongs to the module based on its projection length on the current estimate of the basis. Naturally, it should give higher weights to genes closer to the estimated module subspace. The exact form of the weight function is discussed in the next sub-section. With the weights, the objective function is defined on all genes. We find ***B ***by maximizing the sum of the squared weighted projection lengths, with the constraint that the column vectors of ***B ***form an orthonormal basis.

(2) $$\begin{array}{c} B=(\beta_{1},\beta_{2},...,\beta_{k})=argmax\;{\sum_{i=1}^{p}\left(w_{i}l_{i}\right)}^{2}\\ \text{Subject}\;\text{to}\;\;\left\|\beta_{j}\right\|=1,\;\forall j\\ \beta_{j}⊥\beta_{m,}j\neq m \end{array}$$

With a modular system, we expect the objective function to have multiple local optima, each major local optimum corresponding to one module. Our goal is to seek out a collection of major local optima.

### Weight functions

In this study, we examine two forms of weight function. The first is a sigmoid function.

(3) $$w_{i}=1-\frac{1}{1+e^{\varphi (l_{{}_{i}}-\delta )}}$$

The parameter φ defines the steepness of the curve. When its value is large enough, the shape of the sigmoid function approaches a step function. We can always use a large φ, e.g. 50, to achieve strong contrast between the two groups of genes. When φ is large enough, further increasing its value brings little change to the shape of the curve.

The parameter *δ *defines the inflection point of the sigmoid curve. It is the critical parameter that defines which genes contribute to the estimation of the basis of the module. We find this parameter by considering the distribution of projection length of the null genes - genes not belonging to the module, hence limiting the amount of contribution of such genes.

The parameter *δ *can be determined using the *F *distribution. Based on the theory of linear least squares [22], for genes not belonging to the module, i.e. independent from the basis of the module, the F-statistic,

(4) $$F=\frac{l^{2}}{k}\times \frac{n-k-1}{1-l^{2}}$$

where *n *is the number of samples, and *k *is the dimensionality of the subspace, follows the *F_k, n-k-1 _*distribution. Using a stringent alpha-level cutoff, e.g. 0.001 to account for the large number of genes, we can find the corresponding cutoff in projection length.

(5) $$\delta =\sqrt{\frac{kF_{1-\alpha ,k,n-k-1}}{(n-k-1)+kF_{1-\alpha ,k,n-k-1}}}$$

Because the sigmoid function gives high weight to genes that belong to the module (*l > δ*) and very low weight to genes that do not (*l < δ*), it is the most intuitive for defining modules.

The second weight function is a simple linear weight,

(6) $$w_{i}=l_{i}$$

With this simple weight function, there is no need to pre-specify what projection length corresponds to genes belonging to the module. On the other hand, genes irrelevant to the module can still contribute to the basis selection to a small extent.

### The algorithm for finding the latent factors when the dimensionality of the subspace is known or assumed

Here we present an EM-like iterative algorithm for the optimization, which accommodates both, and potentially other, weight functions. In this section we assume *k *is fixed. The selection of *k *is discussed in subsequence sections. The algorithm iterates between finding the *w's *and the ***β***'s.

When the *w's *are fixed, we first shrink the expression vector of each gene,

(7) $$g_{i}^{*}=w_{i}g_{i}$$

and denote the new weighted expression matrix G*. The objective function is maximized by taking the first *k *right singular vectors of G*. This is because the objective function can be written as,

(8) ∑i=1p     (wili)2=∑i=1p     wi2∑j=1k     (gi'βj)2=∑i=1p     ∑j=1k     (wigi'βj)2

which is the sum of squares of the projection of the weighted data onto the *k*-dimensional subspace.

When the ***β***'s are fixed, every gene is given a weight based on its projection length in the subspace (eq. 3 or eq. 6). We can iterate between finding the ***β***'s and finding the *w's *until convergence:

**Algorithm 1**. Finding ***B ***when *k *is fixed.

(A) Initiate the ***β***'s using *k *randomly selected orthonormal vectors.

(B) Find the latent factors of the module. Iterate:

(B.1) Find the projection length of each gene.

(B.2) Find the weight of each gene (eq. 3 or eq. 6).

(B.3) Multiply each expression vector with its weight.

(B.4) Perform singular value decomposition on the weighted data matrix. Retain the *k *right singular vectors as the new ***β***'s.

(B.5) Perform linear regression of every new ***β ***against all the *k ****β***'s from the previous iteration. If *k *minus the sum of the *R^2 ^*is less than a pre-determined threshold, which means the subspace changes very little in the current iteration, stop the iteration and go to step (C). Otherwise return to step (B.1).

(C) Module membership determination. For every observed projection length l*, we compute the corresponding *F *statistic F* using equation 4. Find:

FDR(l*)=(p × prob(F≥F*))/#(genes with projection length≥l*),

where *p *is the number of genes in the matrix. This is a conservative FDR estimate because we use the count of all genes in the place of the count of null genes. Find the cutoff value *η *- the smallest l* that achieves FDR less than a pre-specified threshold, and assign all the genes with equal or larger projection length to the module.

(D) If *k *>*1*, rotate the basis with oblique rotation, using only loadings from genes with projection length ≥ *η*.

In step (B.2), when using the sigmoid weight function, we initially use a small φ value such as φ* = *φ*/10, and slowly increase at φ* each iteration, until the target *φ *value is reached. The initial smaller φ values results in smaller penalty to genes with short projection length, which allows the algorithm a larger search space.

### Convergence of the algorithm

For the linear weight function, we can show that the value of the objective function is non-decreasing in the iterations proposed in Algorithm 1. From iteration *(t-1) *to *(t)*, the first step is the SVD of the weighted expression matrix. The weight is simply *l_i_*^(*t*-1) ^for gene *i*. It follows from the property of SVD that the first *k *right singular vectors maximize the sum of squares of the projection lengths. With all the row vectors shrunken by a factor of *l_i_*^(*t*-1)^, we have:

(9) $$\sum_{i=1}^{p}\left[l_{i}^{\left(t-1\right)}l_{i}^{\left(t\right)}\right]2\;\;\;\geq \;\;\sum_{i=1}^{p}\left[l_{i}^{\left(t-1\right)}l_{i}^{\left(t-1\right)}\right]2$$

This is true because the left hand side of the inequality represents the sum of squares of the projection lengths using the singular vectors, and the right hand side represents the sum of squares of the projection lengths using another non-optimal basis.

By rearranging (9), we have

(10) $$A=\sum_{i=1}^{p}\left(l_{i}^{\left(t-1\right)}\right)2\left[{\left(l_{i}^{\left(t\right)}\right)}^{2}-{\left(l_{i}^{\left(t-1\right)}\right)}^{2}\right]\geq 0$$

Next we examine the re-weighting step. Now for every gene, we re-assign the weight to be *l_i_*^(*t*) ^. We hope to show that

(11) $$\sum_{i=1}^{p}\left[l_{i}^{\left(t\right)}l_{i}^{\left(t\right)}\right]2\;\;\;\geq \;\;\sum_{i=1}^{p}\left[l_{i}^{\left(t-1\right)}l_{i}^{\left(t\right)}\right]2$$

This is equivalent to showing

(12) $$B\;=\sum_{i=1}^{p}\;\left(l_{i}^{\left(t\right)}\right)2\;\left[\left(l_{i}^{\left(t\right)}\right)2\;-\left(l_{i}^{\left(t-1\right)}\right)2\;\right]\;\;\;\geq \;\;0$$

We subtract *A *from *B*,

(13) $$B\;-A\;=\sum_{i=1}^{p}\;\left[\left(l_{i}^{\left(t\right)}\right)2\;-\left(l_{i}^{\left(t-1\right)}\right)2\;\right]2\;\;\;\geq \;\;0$$

Then because of (10), we have *B≥0*. Hence (11) is true. Combining (9) and (11), we have

(14) $$\sum_{i=1}^{p}\;\left[l_{i}^{\left(t\right)}l_{i}^{\left(t\right)}\;\right]2\;\;\;\geq \;\;\sum_{i=1}^{p}\;\left[l_{i}^{\left(t-1\right)}l_{i}^{\left(t-1\right)}\;\right]2\;$$

Thus we have shown that with every iteration, the value of the objective function is non-decreasing. Hence convergence to a local optimum is guaranteed.

For the sigmoid weight function, this property doesn't hold. Intuitively, with this weight function, the step of finding the *w*'s can be seen as defining module membership of each gene. The iteration is between defining the members and estimating the subspace. In practice, the algorithm with sigmoid weight converged in all the simulations and real data analyses we performed.

### A forward - selection procedure for the automatic determination of *k*

The number of dimensions *k *could be different for different modules. In order to automatically select *k *and the corresponding basis, we describe a forward selection procedure. The procedure is based on the fact that factors within the same module co-regulate some of the genes. Thus when a subset of the factors in a module are found, the residuals of the genes belonging to the module, after fitting to the found factors, provide information regarding the factors that are not yet found.

**Algorithm 2**. The forward selection procedure for the detection of a single module.

(1)Set *k = 1*. Use Algorithm 1 to find ***β_1_***. Currently ***B ***contains only ***β_1_***. Exit if the proportion of genes associated with ***β_1_***, as determined in step (C) of Algorithm 1, is larger than a threshold, e.g. 50%, in which case ***β_1 _***is considered a global factor.

(2)Iterate:

(2.1) Using the current ***B ***matrix, apply the procedure in step (C) of Algorithm 1 to find the genes belonging to the current estimated module. Let the corresponding projection length cutoff be *η*.

(2.2) Select genes belonging to the module, and find their residuals. Multiply the residuals by $1/\sqrt{1-\eta^{2}}$ to restore the range of the residuals to 0[1]. This is done because we make no prior assumption about the relative regulation strength from each hidden factor.

(2.3) Using only the normalized residuals from (2.2), apply Algorithm 1 with *k = 1*, to find the next basis ***β'***.

(2.4) Using all the genes, apply the procedure in step (C) of Algorithm 1 to determine the set of genes that are associated with the newly found basis ***β'***. Use the hypergeometric test to determine if this set of genes significantly overlap with the genes associated with ***B***. If the test result is significant, add the new basis to the ***B ***matrix, and return to step (2.1); else, abandon ***β' ***and go to step (3).

(3) If more than one ***β's ***are found, rotate the basis with oblique rotation, using only loadings from genes with projection length >*η*, which is found in step (2.1).

In step 2.4, because an FDR level is used in determining genes associated with the module (Step C, Algorithm 1), the hypergeometric test is adjusted for the existence of the false-positives in a conservative manner. Assuming the count of genes associated with B is *m_1_*, the count of genes associated with ***β' ***is *m_2_*, the overlap is *r*, and the FDR cutoff is *λ*, we use *m*'_1 _= *ceiling*(*m*_1_(1-*λ*)), *m*'_2 _= *ceiling*(*m*_2_(1-*λ*)), and *r*'= *floor*(*r*(1-*λ*)^2^) for the calculation of the hypergeometric p-value,

$$P=\sum_{l\geq r^{\prime }}\;\;\;\;\frac{\left({}_{m_{2}^{'}-l}^{p-m_{1}^{'}}\right)\left({}_{l}^{m_{1}^{'}}\right)}{\left({}_{m_{2}^{'}}^{p}\right)},$$

where *p *is the number of genes in the data.

We can iterate Algorithm 2 to find a series of modules. The overall workflow is presented in Additional file 1: Figure S1. The number of genes assigned to the module is used as the stopping criterion. In a modular system, modules can be of different sizes. The number of genes assigned to the module can be seen as equivalent to the percentage of variance explained in the PCA setting. When the number of genes in the newly found module is smaller than a threshold, the iteration is stopped.

**Algorithm 3**. Finding a series of modules from a dataset.

Iterate:

(1)Find a module using Algorithm 2.

(2)If the number of genes in the module is smaller than a threshold, end the iteration. Else, take one of the following routes:

(2.a) Remove all genes assigned to the module from the data matrix, return to step (1);

Or alternatively,

(2.b) For each gene, keep the residual by subtracting the projection onto the basis of the module, return to step (1).

### The overall factor model

After finding a collection of ***B ***matrices, we consider all the ***β***'s as latent variables, each of which governs a subset of genes. We can combine them into an overall factor model with a sparse loading matrix to interpret the gene expression. Let *K *be the total number of ***β***'s found, ***F ***be the row-combined factor matrix of all the ***β***'s, ***L ***be the loading matrix, and ***E ***be the unexplained expression, we have a factor model,

$$G_{p\times n}=L_{p\times K}F_{K\times n}+E_{p\times n}$$

The values in ***L ***can be filled in two ways. The first is by performing linear regression of each gene against the factors of the modules the gene is assigned to. The regression is necessary because the factors are rotated and potentially non-orthogonal to each other. Alternatively, we can perform regularized regression of each gene against all the factors. In this report, we used lasso [23] with BIC model selection to determine the factors associated with each gene.

### Simulation study

MLSA was compared to PCA, ICA, factor analysis with oblique rotation, gene shaving [15], and sparse principal component analysis (SPCA) [19] through penalized matrix decomposition [24]. For SPCA, parameter selection was done using cross-validation as provided in the PMA package [24]. Four modes of the MLSA method were tested in combination with the forward-selection scheme: (1) linear weight; removing genes belonging to the module after finding each B matrix; (2) linear weight; retaining the residuals from all genes after finding each B matrix; (3) sigmoid weight; removing genes belonging to the module after finding each B matrix; and (4) sigmoid weight; retaining the residuals from all genes after finding each B matrix.

We considered two classes of latent factor models. The first was the modular system, in which a number of modules exist. Each module contained a subset of genes controlled by module-specific latent factors. Every gene could only belong to one module. Different levels of within-module loading sparsity were considered. The second was the global sparse factor model, in which the latent factors controlled all genes through a sparse loading matrix. Four types of input signals were used for the hidden factors - Gaussian, sine wave, square wave, and sawtooth wave (Additional file 1: Figure S2). A number of scenarios belonging to the following four classes were simulated (Table 1): (1) modular latent structures with hidden factors randomly drawn from the four types; (2) modular latent structures with Gaussian hidden factors; (3) global sparse latent structures with hidden factors randomly drawn from the four types; (4) global sparse latent structures with Gaussian hidden factors. From every possible combination of the parameters (Table 1), 100 simulated data matrices were generated and analyzed.

**Table 1:** Simulation settings.

Parameters	Values	
**Modular factor model**		

Type of hidden factors	Gaussian, mixed	

Number of samples	100	

Number of modules	5	10

Number of genes per module	200	100

Maximum number of factors per module	4	3

Minimum number of factors per module	1	

% non-zero loadings within module	40%, 70%, 100%	

Number of pure noise genes	200, 1000	

Signal to noise ratio	0.5, 1, 2	





**Global sparse factor model**		

Type of hidden factors	Gaussian, mixed	

Number of samples	100	

Number of genes	2500 ^#^	

Number of factors	20	

Average number of factors governing each gene	0.5, 1, 2, 5, 10, 20	

Signal to noise ratio	0.5, 1, 2	

^# ^2000 potentially governed by the factors, 500 pure noise genes.

Within every module, we separately constructed the loading matrix and the matrix of factor scores. The sparsity of the loading matrix was achieved by drawing samples from the binomial distribution. Once the non-zero positions in the loading matrix was determined, for every simulated gene, if there were *m *controlling factors, we divided [0, 1] into *m *regions by drawing *(m-1) *samples from the uniform distribution between 0 and 1. We then used the sizes of the regions as the loadings for the gene. Half of the loadings were then multiplied by -1 to generate negative loadings. The factor scores were generated one factor at a time. When all the four types of factors were used, we first randomly drew the factor type. Then for the non-Gaussian factors, the periodicity *τ *was drawn randomly from [20, 40], and subsequently the phase shift was drawn randomly from [0, *τ*]. Simulated expression matrix of the module was then generated by multiplying the loading matrix with the factor matrix. The simulated matrices from all modules, together with some pure noise genes generated from the Gaussian distribution, were row-combined into a single data matrix. For the global sparse factor model, the matrix was generated as data containing a single module. As the last step, noise generated from the Gaussian distribution was added to the simulated expression matrix.

Among the methods being compared, only MLSA could assign the identified factors into modules. In order to compare the performance, we used the information of the true hidden factors to group the identified factors. Given a simulated data matrix generated from a total of *K *true hidden factors, we allowed each method to find up to *1.5 × K *factors. Notice that *K *is the combined factor count from all modules in the data. In the modular setting, the hidden factors formed groups. In the global sparse factor model, each hidden factor belonged to its own group. First, we performed linear regression of every identified factor against each hidden factor group, and recorded the multiple R^2^. The identified factor was then assigned to the group yielding the largest R^2^. The *K *identified factors with the largest R^2 ^values were retained for the next step. Secondly, we performed linear regression of every true hidden factor against the identified factors assigned to its group, and recorded the multiple R^2 ^as the level of recovery of the true hidden factor. After repeating the simulation from every parameter setting 100 times, we compared the methods by the distribution of the multiple R^2 ^values. The ideal method should yield multiple R^2 ^values close to one.

## Results

### Simulation results

For the modular latent structure model, a total of 72 scenarios were simulated, and for the global sparse factor model, a total of 36 scenarios were simulated (Table 1). Representative results are shown in the main text. More results are in the Additional file 1.

**Figure 1:**
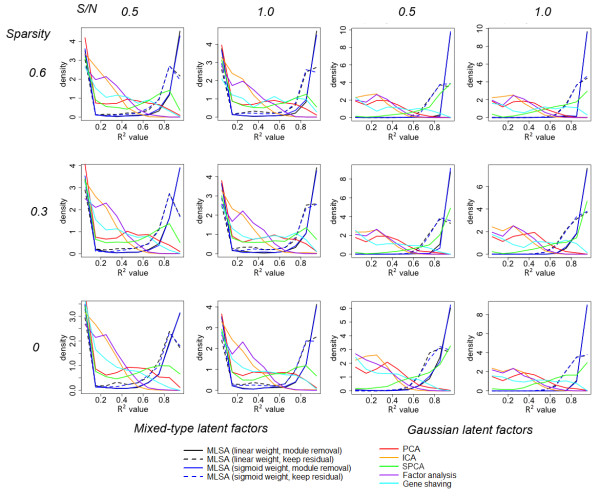
**Simulation results from modular latent structure models**. In every simulation, 10 modules, each consisting of 100 simulated genes, were generated. The number of latent factors per module was randomly selected between 1 and 3. The latent factors were either independent Gaussian (two right columns), or randomly chosen from a mixture of four types (two left columns). Gaussian random noise was added to achieve different signal to noise ratios (columns), and different levels of within-module sparsity (proportion of zero loadings) were tested (rows). An additional 1000 pure noise genes were generated from the standard Gaussian distribution. Each simulation setting was repeated 100 times. The success of latent factor recovery was evaluated by the R^2 ^values obtained by the regression of each latent factor against the identified factors assigned to the module to which the latent factor belongs. The relative frequencies (10 equal-sized bins between 0 and 1, equivalent to the histogram) of the R^2 ^values are plotted.

Figure 1 shows part of the results from simulated modular latent structure models. In all the scenarios, the data contained 10 modules with 100 genes per module. Every module was governed by 1 to 3 (randomly chosen) latent factors. Another 1000 pure noise genes were also included. The two left columns of the subplots are scenarios in which the hidden factors were drawn from four possible types, and the two right columns are scenarios where the hidden factors were drawn from the standard Gaussian distribution. The columns of the subplots correspond to different signal-to-noise ratios, and the rows of the subplots correspond to different levels of within-module sparsity (proportion of zero loadings). For example, the sub-plot at the top-right corner corresponds to the scenario in which 60% of the within-module loadings were zero, and signal variance is equal to that of noise variance.

In all the scenarios, the linear weight and sigmoid weight performed similarly. When the latent variables were all from the standard Gaussian distribution (Figure 1, right panels), MLSA using module removal recovered the hidden factors nearly perfectly (black/blue solid lines). Using residual retention mode, the fidelity of factor recovery suffers (dashed lines), because some hidden factors are not entirely orthogonal to each other. Still, if we consider R^2^≥0.49 (coefficient of multiple correlation ≥ 0.7) as good recovery, then at least 98% of the hidden factors were recovered. SPCA showed very strong performance (green line), in many cases approaching that of MLSA, recovering 84~93% of the hidden factors. Gene shaving recovered 24~49% of the hidden factors. As expected, the non-sparse global methods PCA, ICA and factor analysis performed much worse.

When the latent variables were generated from a mixture of four types of signals (Figure 1, left panels), the percentage of hidden factor recovery was much lower. At the cutoff of R^2^≥0.49, MLSA recovered 59~64% of the hidden factors, while SPCA recovered 43~47%, and gene shaving recovered 18~43%. Interestingly, among the global methods, PCA showed much stronger performance compared to its own performance in the Gaussian hidden factor scenarios, recovering 28~32% of the hidden factors (red line). One interesting characteristic of MLSA is that the latent factors were either recovered with high fidelity, or totally missed. This can be explained by the fact that the method only seeks strong signals from subsets of the genes.

**Figure 2:**
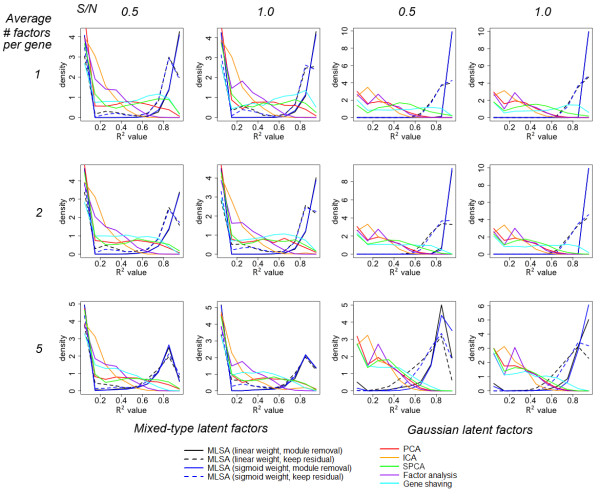
**Simulation results from sparse global latent structure model**. In every simulation, 2000 simulated genes were generated from a latent variable model with 20 latent factors. The latent factors were either independent Gaussian (two right columns), or randomly chosen from a mixture of four types (two left columns). Gaussian random noise was added to achieve different signal to noise ratios (columns), and different levels of sparsity were tested (rows). An additional 500 pure noise genes were generated from the standard Gaussian distribution. Each simulation setting was repeated 100 times. The success of latent factor recovery was evaluated by the R^2 ^values obtained by the regression of each latent factor against the identified factors that are most correlated with it. The relative frequencies (10 equal-sized bins between 0 and 1, equivalent to the histogram) of the R^2 ^values are plotted.

Next we explored the ability of MLSA to recover latent factors when the true model was a sparse global latent structure, instead of modular structure (Figure 2). With Gaussian hidden factors, the results were similar to the modular scenarios when the sparsity is high (average # factors/gene = 1 or 2). A clear deterioration was seen when the average number of factors per gene reached 5. Nonetheless, MLSA still recovered more than 90% of the factors at the cutoff of R^2^≥0.49. The performance of SPCA (11~34% recovery) is not as competitive as in the modular structure scenarios, falling behind gene shaving (19~55% recovery). With mixed-type hidden factors, MLSA recovered 47~61% of the hidden factors, while SPCA recovered 26~33% and gene shaving recovered 17~51%. Again we observed stronger performance of PCA compared to its own performance in the Gaussian hidden factor scenarios.

Overall, the results showed that MLSA was able to recover most latent factors when the factors were generated independently from the Gaussian distribution. When the factors were generated from a mixture of four types, a portion of the true factors were missed by MLSA. Still MLSA performed much better than the other methods tested.

### The yeast cell cycle data

The Spellman cell cycle data consists of four time-series, each covering roughly two cell cycles [25]. The array data consists of 73 conditions and 6178 genes. A number of cell-cycle related genes exhibited strong periodicity in expression. Because of phase differences, the cell cycle related genes cannot be easily summarized by clusters [9].

**Figure 3:**
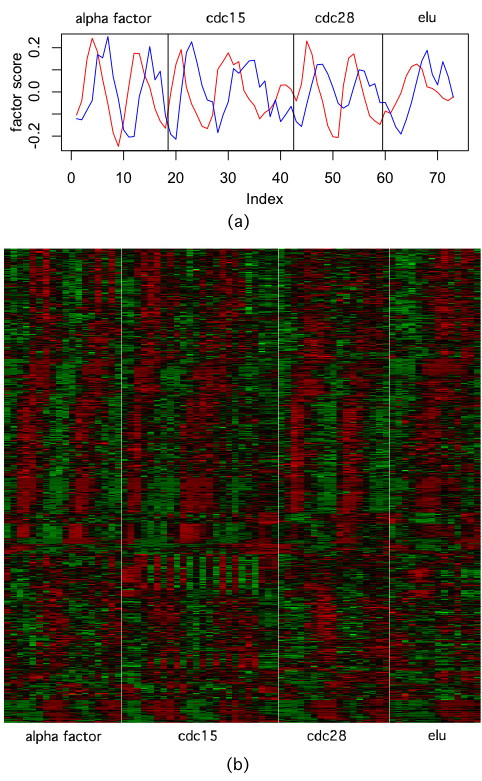
**MLSA results from the yeast cell cycle data**. (a) The factor scores of the module that contains 2 sinusoidal factors; (b) heatmap of all the genes belonging to the module. The rows are genes re-arranged by hierarchical clustering with average linkage; the columns are arrays arranged according to the time series.

We applied MLSA to the cell cycle data as a whole, in order to discover common patterns across the four time series. The results described here were obtained using the sigmoid weight function. Because of the existence of strong global factors, we used the version of MLSA that retains the residuals from all genes after finding each B matrix. Aside from 11 single factors, MLSA found two modules each consisting of two factors. One of the modules was made of two signals of strong periodicity (Figure 3a). Although the periodicity values vary across the four time series, the results clearly confirmed that the same set of genes were involved. Heatmap of the genes belonging to the module show clear periodic behavior with different phase shifts (Figure 3b). The results are consistent with the biological knowledge that cell-cycle related genes are activated at different phases of the cell cycle [25]. When other methods used in the simulation were applied to the cell cycle data, it was clear that non-cell cycle-related signals, such as high-frequency oscillation and linear trend, interfered with the signal separation, yielding no single factor that primarily reflected cell cycle alone. In addition, these methods failed to link the genes with similar periodicity but different phase shifts to a single module.

The factors in the other two-factor module didn't show periodic behavior. To analyze the validity of the module, we resorted to functional analysis based on gene ontology (GO) [26]. Among the 415 genes in the module, 132 were involved in the biological process of translation (p-value 2 × 10^-27^), and another 39 genes were involved in other aspects of gene expression. A large number of other biosynthetic and catalytic processes, including amino acids, steroid, alcohol etc, were also significantly over-represented by the genes in the module. A clear functional consistency is observed in this module.

### The NCI-60 cell lines gene expression data

Next we studied the NCI-60 cell lines gene expression data as measured by U133A array [27]. The NCI-60 cell lines are a collection of cell lines from diverse human cancers. The gene expression and drug response of these cell lines have been studied extensively for the elucidation of cancer mechanisms and screening for drugs. The array data consists of 60 samples and 22215 genes.

After finding the factors by MLSA, we performed regularized regression by lasso to select factors for each gene. The BIC criterion was used in conjunction with a p-value cutoff of 1 × 10^-3 ^for factor selection. A total of 12 factors were identified by MLSA. Two of the factors belong to one module, and three other factors belong to another module (Table 2).

**Table 2:** List of modules from the NCI60 data.

Module	# genes associated (out of 22215 genes)	Factor	F-test p-value*
**1**	4238	1	**0.0076**

**2**	3602	2	**6.2 × 10^-12^**
		3	**7.5 × 10^-9^**

**3**	1008	4	0.072

**4**	1314	5	0.14

**5**	796	6	**0.042**
		7	**7.6 × 10^-4^**
		8	**3.0 × 10^-7^**

**6**	304	9	**0.022**

**7**	231	10	**2.9 × 10^-5^**

**8**	188	11	0.34

**9**	134	12	0.054

* ANOVA of factor score against tissue origins of the cancer cell lines.

**Figure 4:**
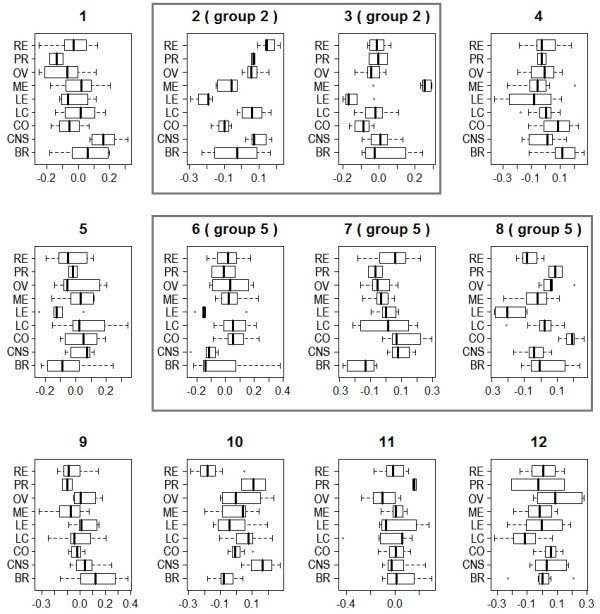
**Boxplots of the factor scores for cancer cell lines from different tissue origin**. Factors belonging to the same module are boxed.

We tested whether each factor was associated with the tissue origin of the tumors by one-way analysis of variance (ANOVA). At the single factor level, six of the factors were significantly associated with the tissue origin of the cancer at the alpha level of 0.01, and two others at alpha level of 0.05 (Table 2). Examination of the boxplots showed some strong differences of factor scores based on tissue origin (Figure 4). Notice that factors in each multi-factor module are unidentifiable and the scores were obtained by oblique rotation [28]. This is because once the module subspace is determined, we can rotate the basis within the subspace and the value of the objective function (eq. 2) doesn't change. This is similar to the situation in exploratory factor analysis.

We further examined the gene lists in the two multi-factor modules through gene ontology. For the two-factor module, over-represented biological processes include mRNA metabolic process (p-value 1.0 × 10^-13^), DNA replication (p-value 0.00018), chromatin modification (p-value 0.00021), blood vessel development (p-value 0.00077), cytoskeleton organization (p-value 0.00077), cell adhesion (p-value 0.0022), apoptosis (p-value 0.0033) and more than 120 other processes, many of which have clear links to tumor development. For the three-factor module, over-represented biological processes include small GTPase mediated signal transduction (p-value 0.0020), RNA splicing (p-value 0.0013) and 14 other processes. Although the functional consistency of this module was not as clear-cut as the other module, we noticed that the module consisted very strong signals separating some cancer types from others (Table 2).

### The squamous cell lung carcinomas data

The third dataset we studied was the squamous cell lung carcinomas data from 129 patients [29]. The array data consists of 130 samples and 22215 genes. Clinical information, including tumor stage, differentiation, survival *etc*. are also available.

MLSA identified a five-factor module, a six-factor module, a three-factor module, two two-factor module and another 18 single factors. By performing Cox proportional hazard regression with survival outcome, and ordered logistic regression with tumor stage or tumor differentiation as outcome, we found that the five-factor module was significantly associated with tumor differentiation (p-value 0.0097). The gene list of this module over-represents many biological processes associated with tumor development, such as cell adhesion (p-value 4.0 × 10^-10^), cell proliferation (p-value 5.6 × 10^-7^), immune response (p-value 6.1 × 10^-7^), response to wounding (p-value 2.1 × 10^-5^), blood vessel development (p-value 6.6 × 10^-4^), and cell migration (p-value 0.0014). One of the two-factor modules was significantly associated with tumor stage (p-value 0.0094). Its genes over-represent processes such as regulation of osteoblast differentiation (p-value 0.0016), bone remodeling (p-value 0.0017), and negative regulation of inflammatory response (p-value 0.0086).

The six-factor module was associated with survival outcome with marginal significance (p-value 0.060). The genes in this module over-represents biological processes in immune response and macromolecule biosynthesis, such as lymphocyte activation (p-value 8.0 × 10^-6^), translational elongation (p-value 1.6 × 10^-15^), post-translational protein modification (p-value 0.0024), and protein amino acid phosphorylation (p-value 0.0012). Another two-factor module associated with differentiation with marginal significance (p-value 0.063). Its genes over-represent processes such as protein metabolic process (p-value 0.001334), regulation of organelle organization (p-value 0.0040), ubiquitin-dependent protein catabolic process (p-value 0.0081), and coenzyme metabolic process (p-value 0.0092). Among the five multi-factor modules, four were associated with clinical outcomes to some extent. Three of the 18 single factors also showed significant associations with the outcomes.

## Discussion

The purpose of the MLSA method is to find a collection of basis, such that each basis explains the expression of a subset of genes well. In a modular system, multiple local optima exist, each corresponding to a module. The MLSA algorithm searches for modules in an iterative manner. The ideal algorithm should find the global optimum in each round. However, this is a difficult task. Using the linear weight function, the MLSA method finds one local optimum at a time. The issue of not necessarily finding the global optimum is alleviated by the purpose of the algorithm - it is intended to find a series of local optima. If the global optimum is missed in one round of search, it could still be discovered in subsequent rounds.

The sigmoid weight function doesn't guarantee the value of the objective function to be non-decreasing. However, it is more intuitive in that genes with small projections (cutoff defined using null distribution) contribute very little to the estimation of the basis, and genes with large projections contribute to the estimation almost equally. The weighting step can be seen as estimating the module membership, and the weighted SVD step estimates the subspace based on the current estimates of module membership. The use of the weight function is mainly justified by our simulation study - the algorithm always converged, and usually within fewer iterations compared to the linear weight function.

The MLSA method seeks subspaces using a projection-based algorithm. When hidden factors highly correlate with each other, their subspaces overlap. MLSA will not be able to separate the highly correlated signals. Rather, the signals will likely be combined into a single factor when identified. In our simulations using mixed-type hidden factors, some factors were correlated due to the characteristics of the wave functions, even though their periodicities and phases were drawn independently. In fact 10% of the absolute correlation coefficients between factors were higher than 0.5, half of which were higher than 0.66. Given that MLSA makes no assumption about signal distributions, the most likely explanation of the worse performance in the mixed-type signal scenarios compared to the Gaussian signal scenarios is the high correlation of the signals.

After finding each module, there are two ways to remove the influence of the module before searching for the next module. MLSA either removes the genes that are members of the module, or takes the residuals of all genes. Which method to choose depends on the characteristics of the data. If the basis of the module influences a large proportion of the genes, which is sometimes observed in real microarray data, taking the residuals is recommended. When using the residuals, the bases of different modules are strictly orthogonal to each other. When using module member removal, the bases of the modules could be weakly correlated. In real biological systems, some input signals, e.g. transcription factor activities, could be correlated [13,14]. In the real data we examined, the correlations between the bases were relatively low. For example, when the method of module member removal was applied on the cell cycle data, the absolute correlations between the factors were all below 0.2.

A number of parameters are involved in the MLSA algorithm. For both linear and sigmoid weight function, an FDR cutoff is needed in order to determine module membership (Algorithm 1), and a cutoff in alpha level for the hypergeometric test needs to be defined in the forward selection of basis (Algorithm 2). Both these parameters carry straight-forward statistical interpretations, and proper levels can be selected by the user. For the sigmoid weight function, two extra parameters need to be set. The first parameter is the shape parameter φ of the sigmoid function (eq. 3). As we discussed in the methods section, when φ is large, the sigmoid function approaches a step function, and further increase in φ has little effect on the shape of the curve. Thus the exact choice is not very critical and a large φ value can always be used. The second is the alpha level for the *F *distribution (eq. 4), which determines the inflection point δ of the weight function (eq. 5). When setting this parameter, it is necessary to consider the issue of multiple testing. Otherwise the contribution from genes unrelated to the module could influence the estimation result, and the identified basis may carry more global information, rather than the information local to the module. This will in turn impact not only the estimation of the current module, but other modules that have not been identified yet.

In the search of a series of modules, the stopping rule is based on the number of genes assigned to the newly found module (Algorithm 3). In a modular system, this parameter can be seen as similar to the percentage of variance explained in the PCA setting. Because modules are discovered in a sequential manner, and the algorithm for basis estimation (Algorithm 1) isn't guaranteed to converge to the global optimum, it is recommended that a very small cutoff value, e.g. 10 genes, is used in the module discovery phase. Then the user can select modules based on the number of genes associated with each module, possibly after re-assigning the loadings through gene-by-gene variable selection.

## Conclusions

In summary, the problem of identifying modular structures without any prior information is a difficult one. The MLSA algorithm utilizes the fact that each module occupies a subspace of much lower dimension. The method seeks subspaces in which a subset of genes have large projections. It performs well in simulations, and generates biologically relevant results from real datasets. An interesting observation is that the method also recovers hidden factors with high fidelity when the true model is a global sparse factor model, which makes it a good choice for the purpose of blind source separation.

## Supplementary Material

Additional file 1 **Supporting Material**. Supporting figures and detailed results of the simulation study. Click here for file
