# Supplementary material for: An exploratory data analysis method to reveal modular latent structures in high-throughput data
Source: BMC Bioinformatics. 2010 Aug 27;11:440. doi: 10.1186/1471-2105-11-440 (PMC2940911; doi:10.1186/1471-2105-11-440)
Supplement: Additional file 1 — Supporting Material. Supporting figures and detailed results of the simulation study. [file 1471-2105-11-440-S1.PDF]

Supporting materials

S.1. Additional Figures.

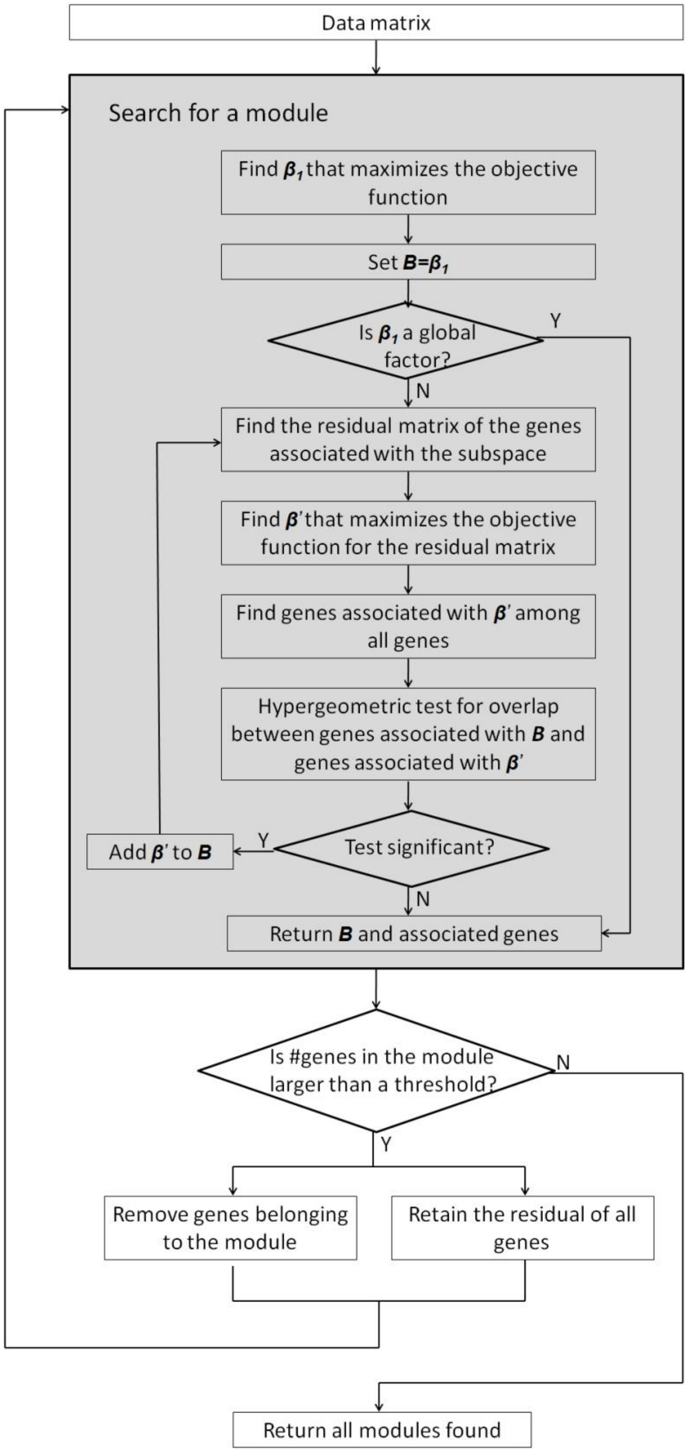

Figure S1. The overall workflow of module detection from a data matrix. The flow chart corresponds to Algorithm 3 in the main text. The grey box corresponds to Algorithm 2 in the main text. The steps of maximizing objective function utilize Algorithm 1 in the main text.

S.2. The simulation study.

1. The setup.

Four types of input signals were used for the hidden factors in the simulations – Gaussian, sine wave, square wave, and sawtooth wave (Figure S2). Several scenarios were simulated:

(1) Modular latent structures with mixed-type hidden factors

(2) Modular latent structures with Gaussian hidden factors

(3) Global sparse latent structure with mixed-type hidden factors

(4) Global sparse latent structure with Gaussian hidden factors

The parameter settings are listed in Table 1 of the main text. In all scenarios 100 samples were simulated. When non-Gaussian factors were used, the periodicity of signal types (2)~(4) were drawn randomly from [20,40], and subsequently the phase shift were drawn randomly from [0, periodicity].

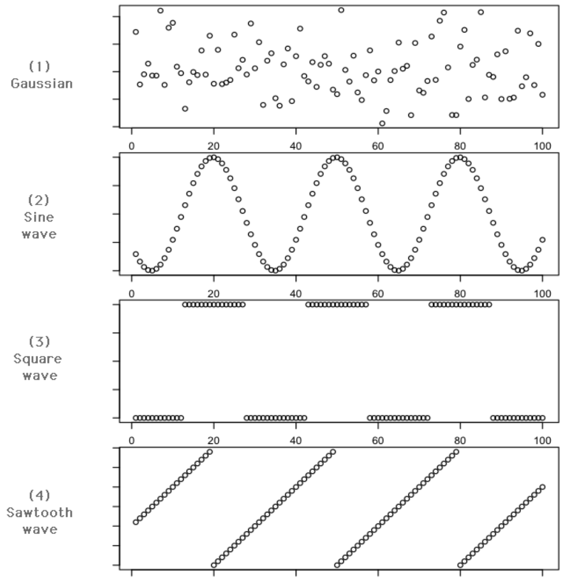

Figure S2. The four types of input signal from which the data were simulated.

For every simulated gene, if there were *m* controlling factors, we divided [0, 1] into *m* regions by drawing *(m-1)* values from the uniform distribution between 0 and 1. We then used the sizes of the regions as the loadings. Half of the loadings were then multiplied by -1 to generate negative loadings. When a certain level of sparsity is used, we randomly selected entries of the loading matrix to be non-zero before assigning the loading values. Simulated expression profiles were then generated by multiplying the loading matrix and the factor matrix. Noise generated from the Gaussian distribution was added to the expression profiles.

2. Methods tested.

Four modes of MLSA were tested:

(1) Linear weight; removing genes belonging to the module after finding each B matrix.
(2) Linear weight; retaining the residuals of all genes after finding each B matrix.
(3) Sigmoid weight; removing genes belonging to the module after finding each B matrix.
(4) Sigmoid weight; retaining the residuals from all genes after finding each B matrix.

Global dimension reduction/ blind source separation methods tested:

(5) Principal component analysis (PCA);
(6) Independent Component Analysis (ICA);
(7) Sparse Principal Component Analysis (SPCA);
(8) Factor analysis with oblique rotation;
(9) Gene shaving. The mean profile of every cluster is considered the single latent factor for that cluster.

3. Evaluation of performance.

Given a simulated gene expression matrix generated from a total of K true hidden factors, we allowed each method to find $1.5\times K$ factors. In the modular setting, the hidden factors form groups, each of which may be of different size. In the global sparse factor model, each hidden factor belongs to its own group. We first assign the identified factors to the closest group, and then find the level of recovery of the hidden factors.

(1) We perform regression of every identified factor against each hidden factor group, and record the multiple R^2 from each regression. The identified factor is then assigned to the group yielding the largest R^2. The K factors with the largest R^2 values are kept.

(2) We perform regression of every hidden factor on the identified factors assigned to its group, and record the multiple R^2 as the level of recovery of the hidden factor.

(3) After repeating the simulation from every parameter setting 100 times, we compare the methods by the distribution of the multiple R^2 values from step 2. The ideal method should yield multiple R^2 values close to one.

4. Results of the simulation.

4.1. Modular latent structures with mixed-type hidden factors.

4.1.1. Five modules, each with 200 genes.

A total of 1000 simulated genes were potentially governed by the latent factors. In the first scenario, we added another 200 pure noise genes, and allowed one to four (randomly chosen) factors to govern each group. MLSA clearly led the performance in recovering hidden factors with high fidelity (Figure S3). On the other hand, when a factor is missed by MLSA, it tends to be missed entirely. When the S/N is low (0.5), better results were obtained with higher within-module sparsity.

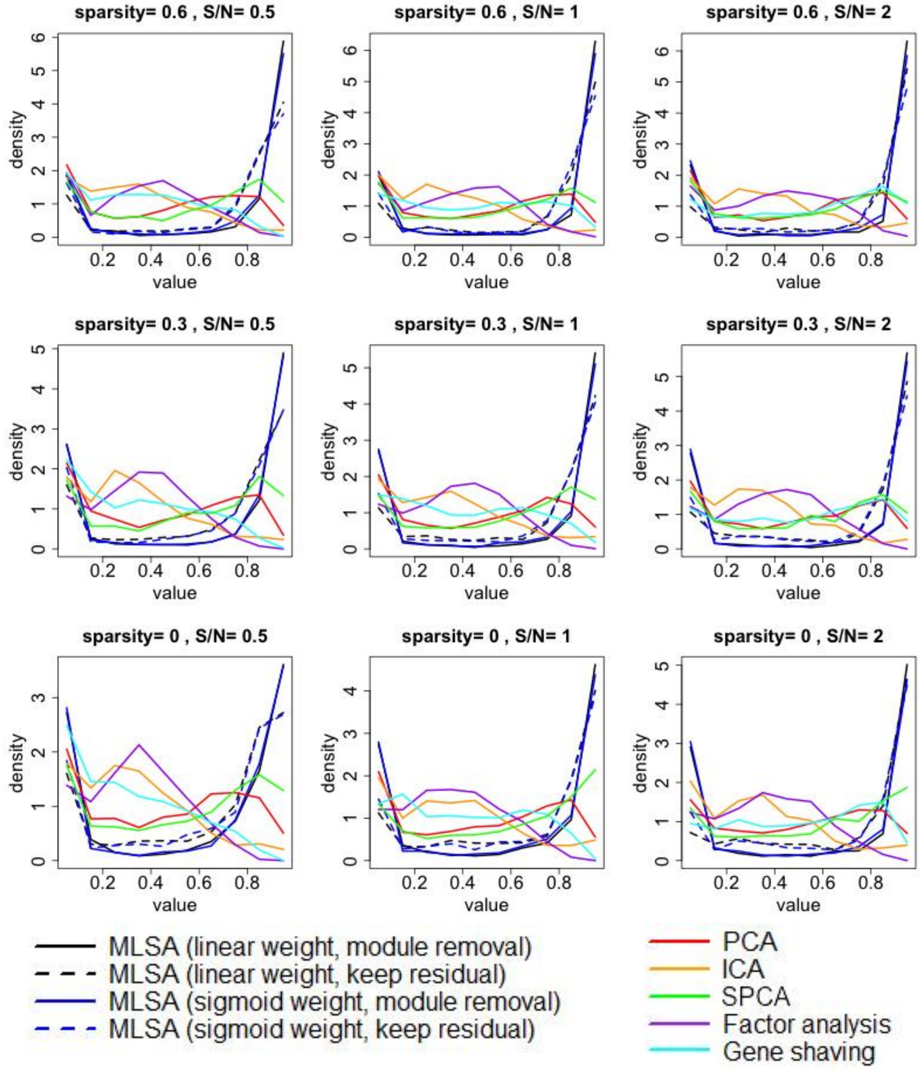

Figure S3. Simulation results from modular latent structure models. In every simulation, 5 modules, each consisting of 200 simulated genes, were generated. The number of latent factors per module was randomly selected between 1 and 4. The latent factors were randomly chosen from a mixture of four types. Gaussian random noise was added to achieve different signal to noise ratio (columns), and different levels of within-module sparsity were tested (rows). An additional 200 pure noise genes were generated from normal distribution. Each simulation setting was repeated 100 times. The success of latent factor recovery was evaluated by the R^2 values obtained by the regression of each latent factor against the identified factors that are most correlated with the module to which the latent factor belongs. The relative frequencies (10 equal-sized bins between 0 and 1) of the R^2 values are plotted.

The number of pure noise genes was then increased to 1000. The impact of this change was small (Figure S4, compared to Figure S3).

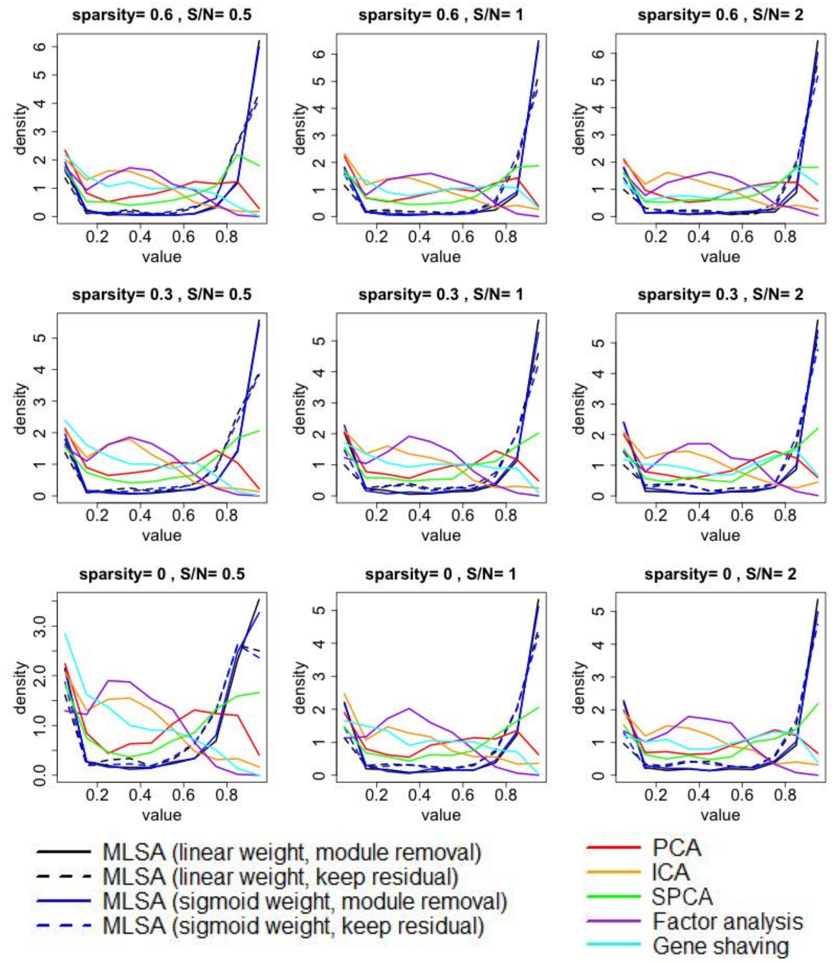

Figure S4. Simulation results from modular latent structure models. In every simulation, 5 modules, each consisting of 200 simulated genes, were generated. The number of latent factors per module was randomly selected between 1 and 4. The latent factors were randomly chosen from a mixture of four types. Gaussian random noise was added to achieve different signal to noise ratio (columns), and different levels of within-module sparsity were tested (rows). An additional 1000 pure noise genes were generated from normal distribution. Each simulation setting was repeated 100 times. The success of latent factor recovery was evaluated by the R^2 values obtained by the regression of each latent factor against the identified factors that are most correlated with the module to which the latent factor belongs. The relative frequencies (10 equal-sized bins between 0 and 1) of the R^2 values are plotted.

4.1.2. Ten modules, each with 100 genes.

In the first scenario, we added another 200 pure noise genes, and allowed one to three (randomly chosen) factors to govern each group. MLSA clearly lead in recovering hidden factors with high fidelity (Figure S5). On the other hand, when a factor is missed by MLSA, it tends to be missed entirely. Possibly because more

latent factors are involved, the results are not as good as those from the 5 module scenario (Figure S5, compared to Figure S3).

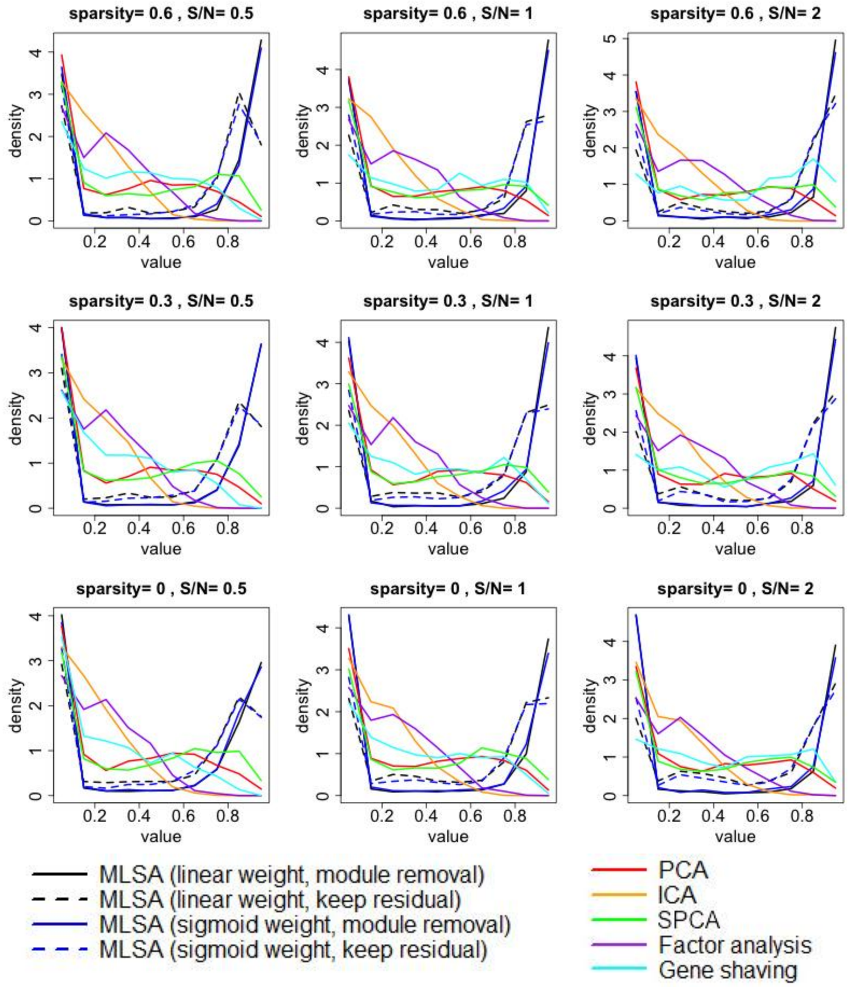

Figure S5. Simulation results from modular latent structure models. In every simulation, 10 modules, each consisting of 100 simulated genes, were generated. The number of latent factors per module was randomly selected between 1 and 3. The latent factors were randomly chosen from a mixture of four types. Gaussian random noise was added to achieve different signal to noise ratio (columns), and different levels of within-module sparsity were tested (rows). An additional 200 pure noise genes were generated from normal distribution. Each simulation setting was repeated 100 times. The success of latent factor recovery was evaluated by the R^2 values obtained by the regression of each latent factor against the identified factors that are most correlated with the module to which the latent factor belongs. The relative frequencies (10 equal-sized bins between 0 and 1) of the R^2 values are plotted.

The number of pure noise genes was then increased to 1000. The impact of this change was small (Figure S6, compared to Figure S5).

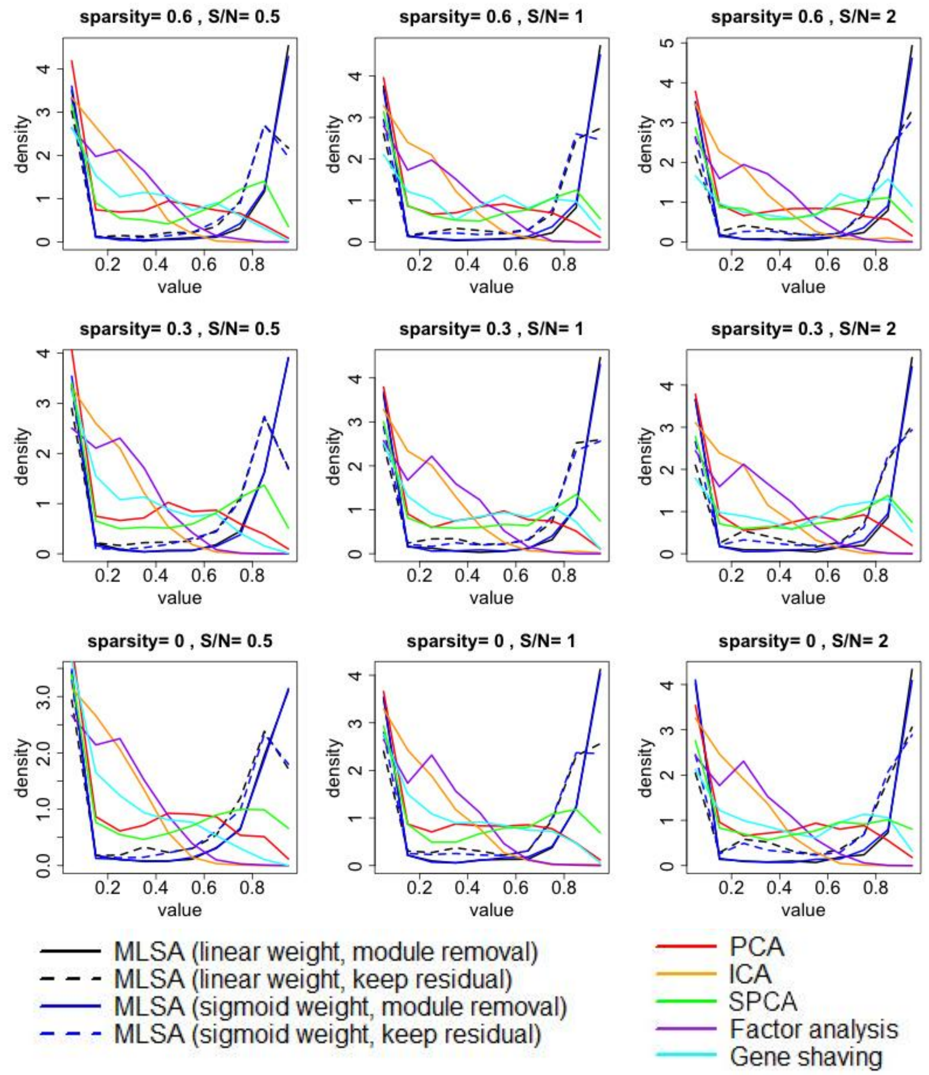

Figure S6. Simulation results from modular latent structure models. In every simulation, 10 modules, each consisting of 100 simulated genes, were generated. The number of latent factors per module was randomly selected between 1 and 3. The latent factors were randomly chosen from a mixture of four types. Gaussian random noise was added to achieve different signal to noise ratio (columns), and different levels of within-module sparsity were tested (rows). An additional 1000 pure noise genes were generated from normal distribution. Each simulation setting was repeated 100 times. The success of latent factor recovery was evaluated by the R^2 values obtained by the regression of each latent factor against the identified factors that are most correlated with the module to which the latent factor belongs. The relative frequencies (10 equal-sized bins between 0 and 1) of the R^2 values are plotted.

4.2. Modular latent structures with Gaussian hidden factors.

4.2.1. Five modules, each with 200 genes.

A total of 1000 simulated genes were potentially governed by the latent factors. In the first scenario, we added another 200 pure noise genes, and allowed one to four (randomly chosen) factors to govern each group. MLSA clearly lead in recovering

hidden factors with high fidelity (Figure S7). MLSA almost recovered all hidden factors.

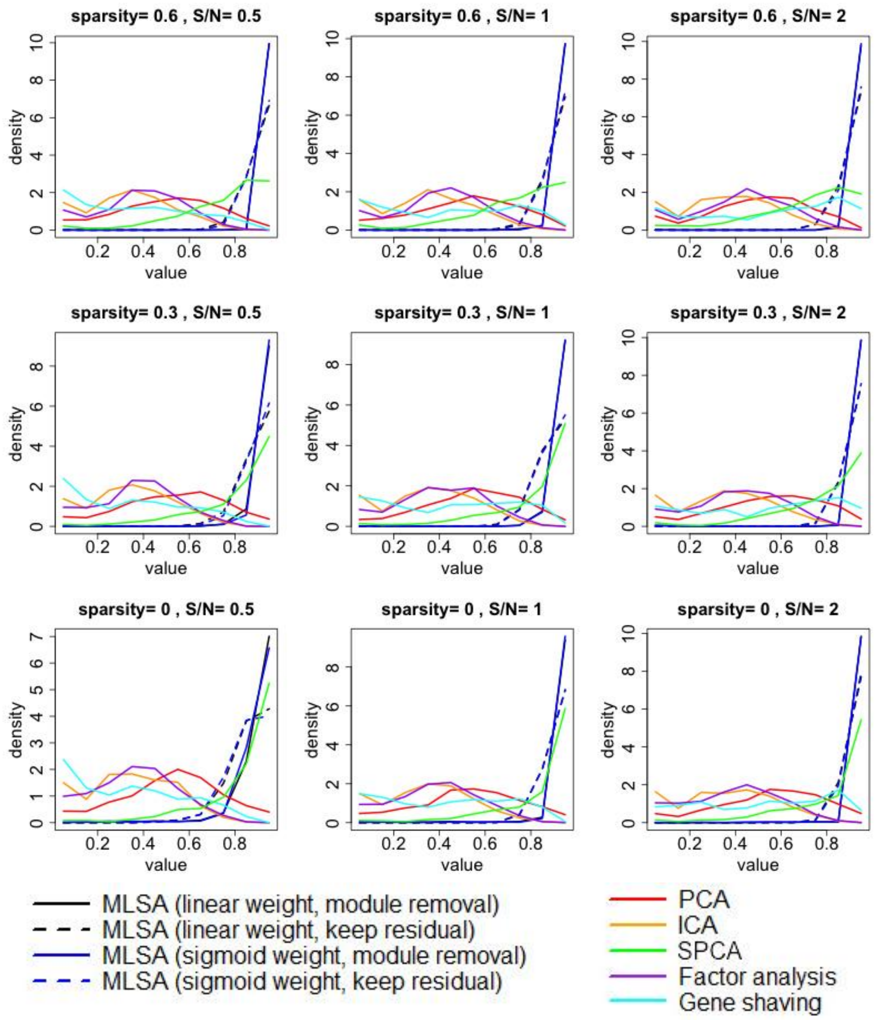

Figure S7. Simulation results from modular latent structure models. In every simulation, 5 modules, each consisting of 200 simulated genes, were generated. The number of latent factors per module was randomly selected between 1 and 4. The latent factors were generated from the Gaussian distribution. Gaussian random noise was added to achieve different signal to noise ratio (columns), and different levels of within-module sparsity were tested (rows). An additional 200 pure noise genes were generated from normal distribution. Each simulation setting was repeated 100 times. The success of latent factor recovery was evaluated by the R^2 values obtained by the regression of each latent factor against the identified factors that are most correlated with the module to which the latent factor belongs. The relative frequencies (10 equal-sized bins between 0 and 1) of the R^2 values are plotted.

Increasing the number of pure noise genes to 1000, MLSA failed to recover a small portion of the latent factors when the S/N was low. Again better performance was obtained with higher sparsity (Figure S8).

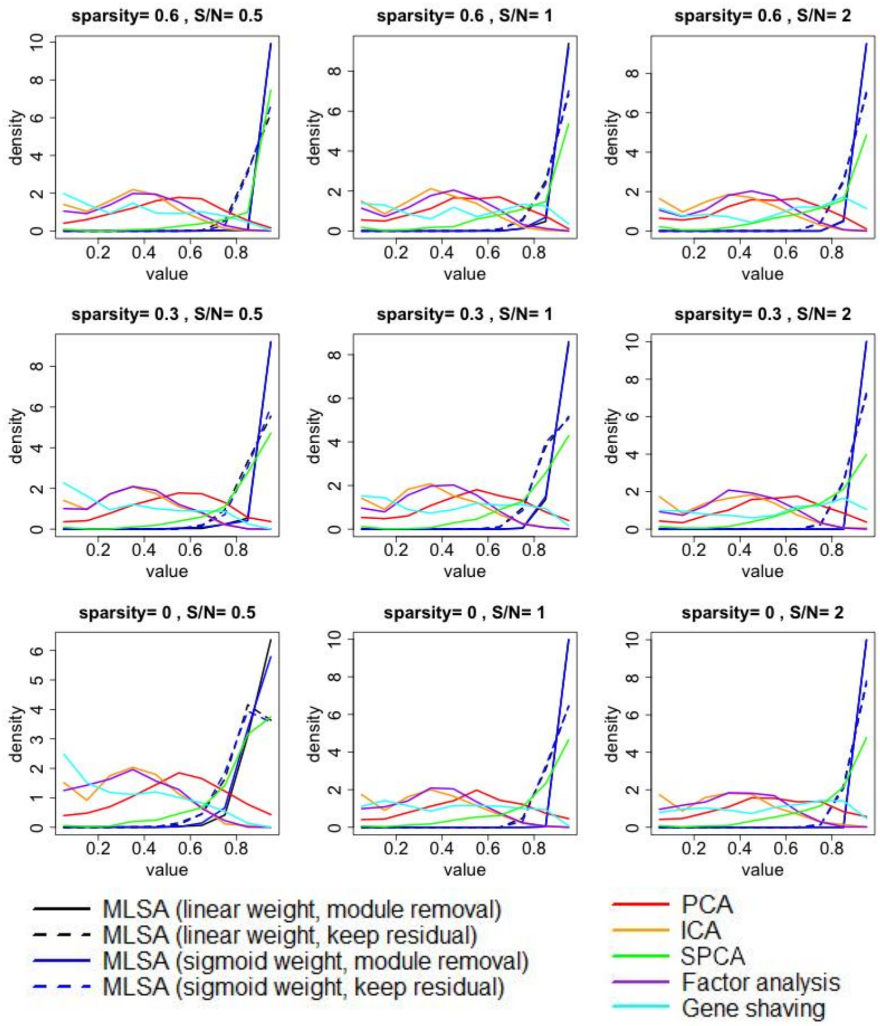

Figure S8. Simulation results from modular latent structure models. In every simulation, 5 modules, each consisting of 200 simulated genes, were generated. The number of latent factors per module was randomly selected between 1 and 4. The latent factors were generated from the Gaussian distribution. Gaussian random noise was added to achieve different signal to noise ratio (columns), and different levels of within-module sparsity were tested (rows). An additional 1000 pure noise genes were generated from normal distribution. Each simulation setting was repeated 100 times. The success of latent factor recovery was evaluated by the R^2 values obtained by the regression of each latent factor against the identified factors that are most correlated with the module to which the latent factor belongs. The relative frequencies (10 equal-sized bins between 0 and 1) of the R^2 values are plotted.

4.2.2. Ten modules, each with 100 genes.

A total of 1000 simulated genes were potentially governed by the latent factors. In the first scenario, we added another 200 pure noise genes, and allowed one to three (randomly chosen) factors to govern each group. MLSA clearly led in recovering hidden factors with high fidelity (Figure S9).

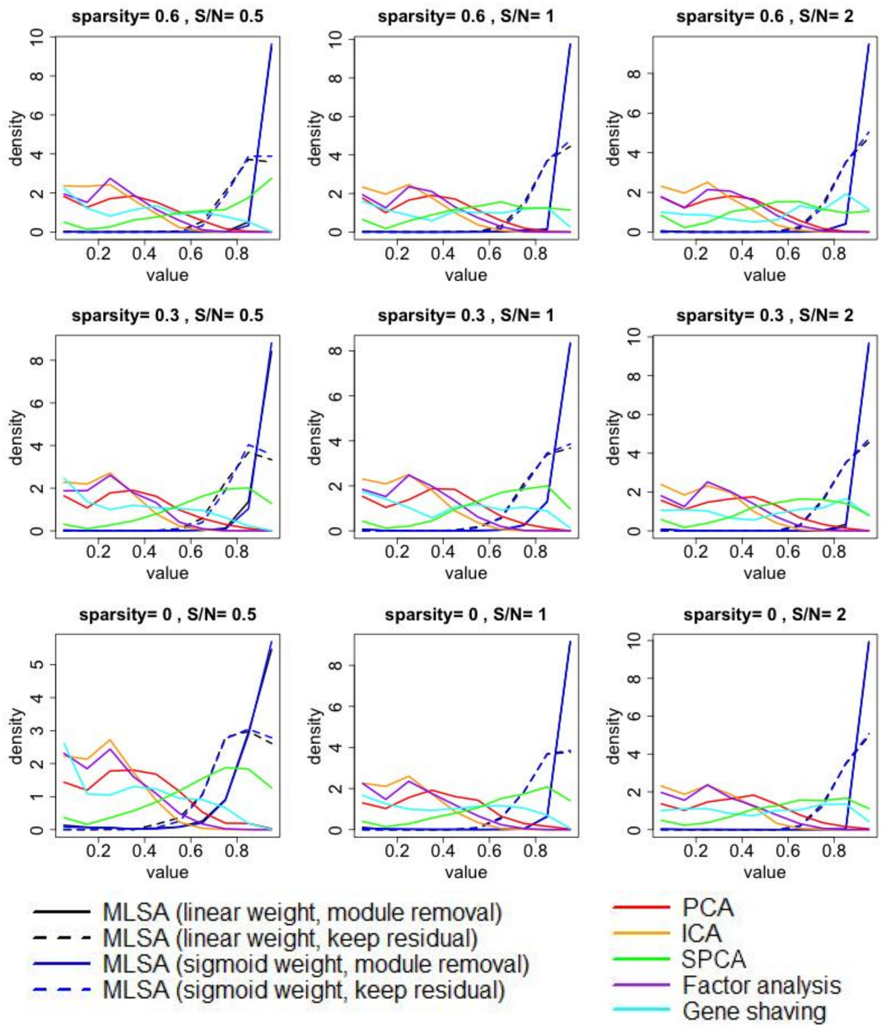

Figure S9. Simulation results from modular latent structure models. In every simulation, 10 modules, each consisting of 100 simulated genes, were generated. The number of latent factors per module was randomly selected between 1 and 3. The latent factors were generated from the Gaussian distribution. Gaussian random noise was added to achieve different signal to noise ratio (columns), and different levels of within-module sparsity were tested (rows). An additional 200 pure noise genes were generated from normal distribution. Each simulation setting was repeated 100 times. The success of latent factor recovery was evaluated by the R^2 values obtained by the regression of each latent factor against the identified factors that are most correlated with the module to which the latent factor belongs. The relative frequencies (10 equal-sized bins between 0 and 1) of the R^2 values are plotted.

When the number of pure noise genes was increased to 1000, the overall performance is slightly impaired (Figure S10 v.s. S9). Yet still at medium to higher S/N, MLSA recovered most latent factors.

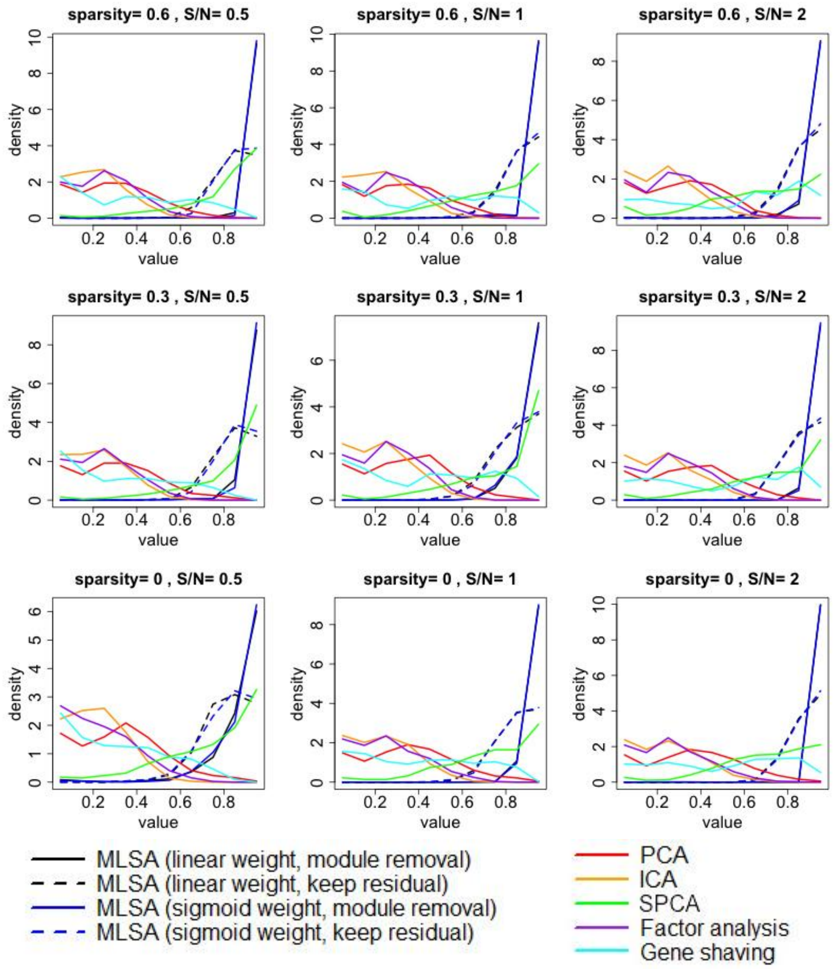

Figure S10. Simulation results from modular latent structure models. In every simulation, 10 modules, each consisting of 100 simulated genes, were generated. The number of latent factors per module was randomly selected between 1 and 3. The latent factors were generated from the Gaussian distribution. Gaussian random noise was added to achieve different signal to noise ratio (columns), and different levels of within-module sparsity were tested (rows). An additional 1000 pure noise genes were generated from normal distribution. Each simulation setting was repeated 100 times. The success of latent factor recovery was evaluated by the R^2 values obtained by the regression of each latent factor against the identified factors that are most correlated with the module to which the latent factor belongs. The relative frequencies (10 equal-sized bins between 0 and 1) of the R^2 values are plotted.

4.3. Global sparse latent structures with mixed-type hidden factors.

In this simulation, 2000 genes were generated from 20 mixed-type latent factors, at various sparsity (average # controling factors per gene) and S/N settings. Another 500 pure noise genes were added. Even though the results were not as good as those from mudular latent structures, MLSA still clearly led in performance in tofhe recovery of true latent factors. The best performance of MLSA was achieved when on average two or less factors control each gene, i.e. >90% sparsity of the loading matrix. MLSA recovered (R^2>0.6) ~80% of the hidden factors under such settings (Figure S11).

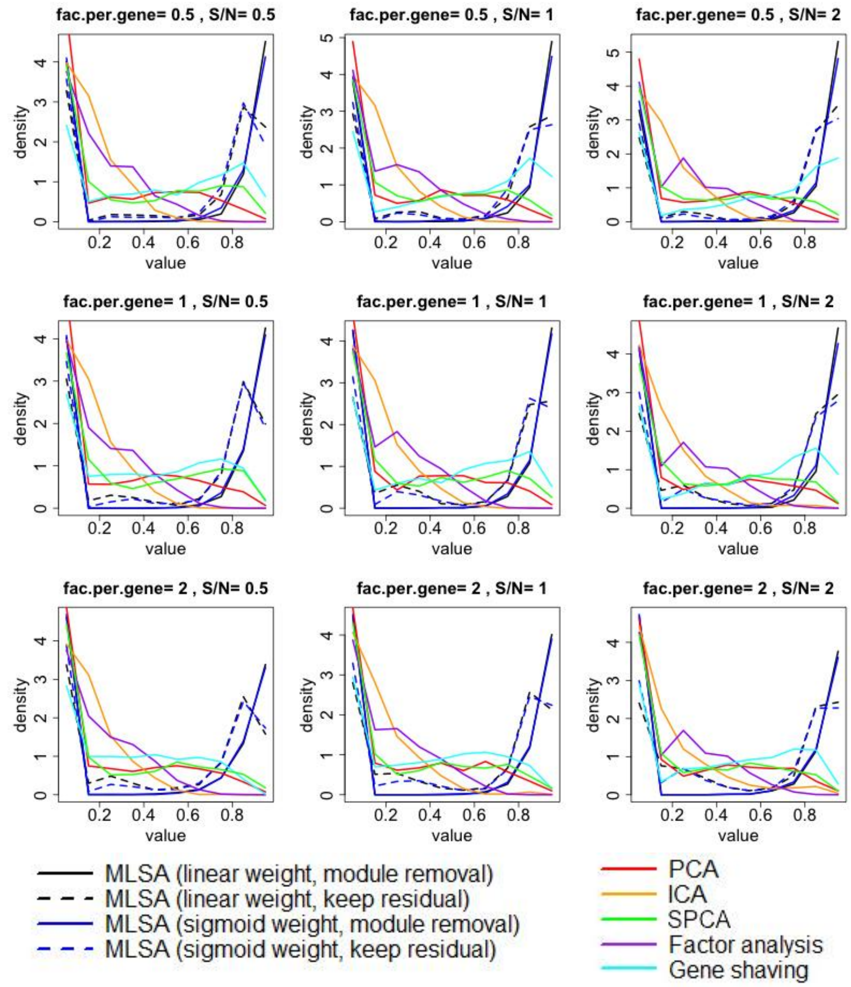

Figure S11. Simulation results from sparse global latent structure model. In every simulation, 2000 simulated genes were generated from a latent variable model with 20 latent factors. The latent factors were randomly chosen from a mixture of four types. Gaussian random noise was added to achieve different signal to noise ratio (columns), and different levels of sparsity were tested (rows). An additional 500 pure noise genes were generated from normal distribution. Each simulation setting was repeated 100 times. The success of latent factor recovery was evaluated by the R^2 values obtained by the regression of each latent factor against the identified factors that are most correlated with it. The relative frequencies (10 equal-sized bins between 0 and 1) of the R^2 values are plotted.

MLSA's performance deteriorates when the loadings became denser, yet it mainains an edge when the average number of factors per gene is up to 10, which is already an irrelevant level in real biological networks (Figure S12).

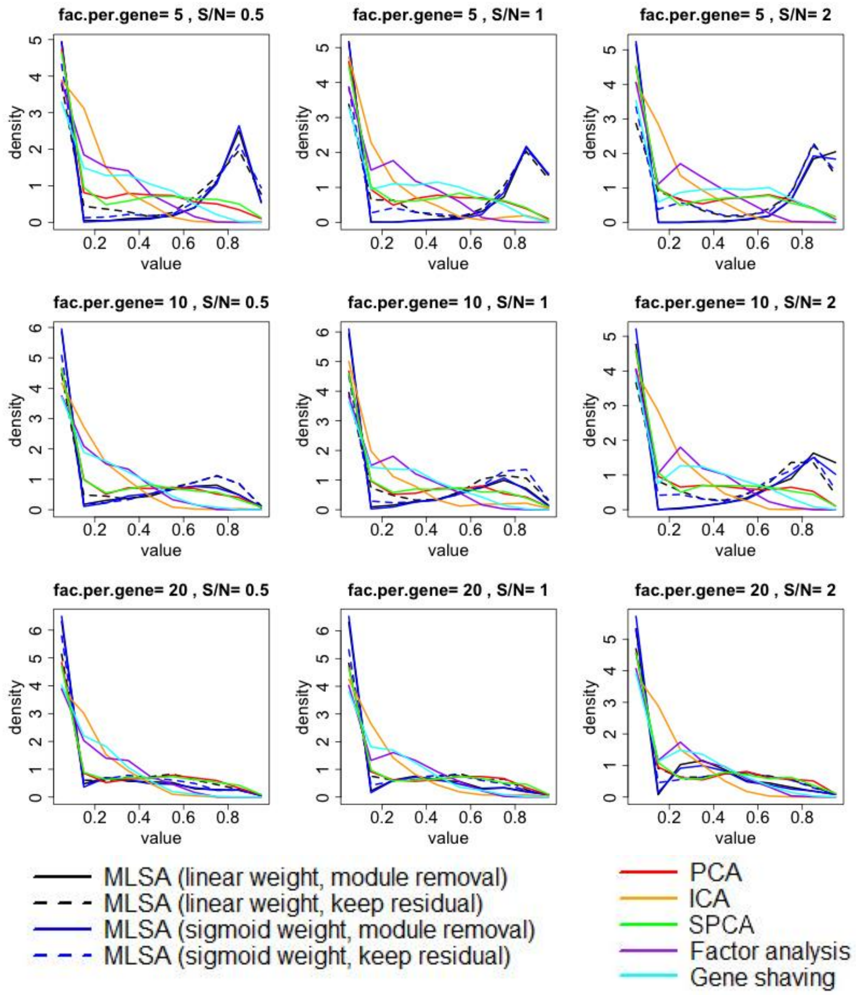

Figure S12. Simulation results from sparse global latent structure model. In every simulation, 2000 simulated genes were generated from a latent variable model with 20 latent factors. The latent factors were randomly chosen from a mixture of four types. Gaussian random noise was added to achieve different signal to noise ratio (columns), and different levels of sparsity were tested (rows). An additional 500 pure noise genes were generated from normal distribution. Each simulation setting was repeated 100 times. The success of latent factor recovery was evaluated by the R^2 values obtained by the regression of each latent factor against the identified factors that are most correlated with it. The relative frequencies (10 equal-sized bins between 0 and 1) of the R^2 values are plotted.

4.4. Global sparse latent structures with Gaussian hidden factors.

In this simulation, 2000 genes were generated from 20 Gaussian latent factors, at various sparsity (average # controling factors per gene) and S/N settings. Another 500 pure noise genes were added. Again the results from Gaussian factor scenarios were much better than those from mixed-type factors. When the average number of factors per gene was between 1 and 5, MLSA (using module removal when a module was identified) recovered almost all of the latent factors (Figures S13, S14).

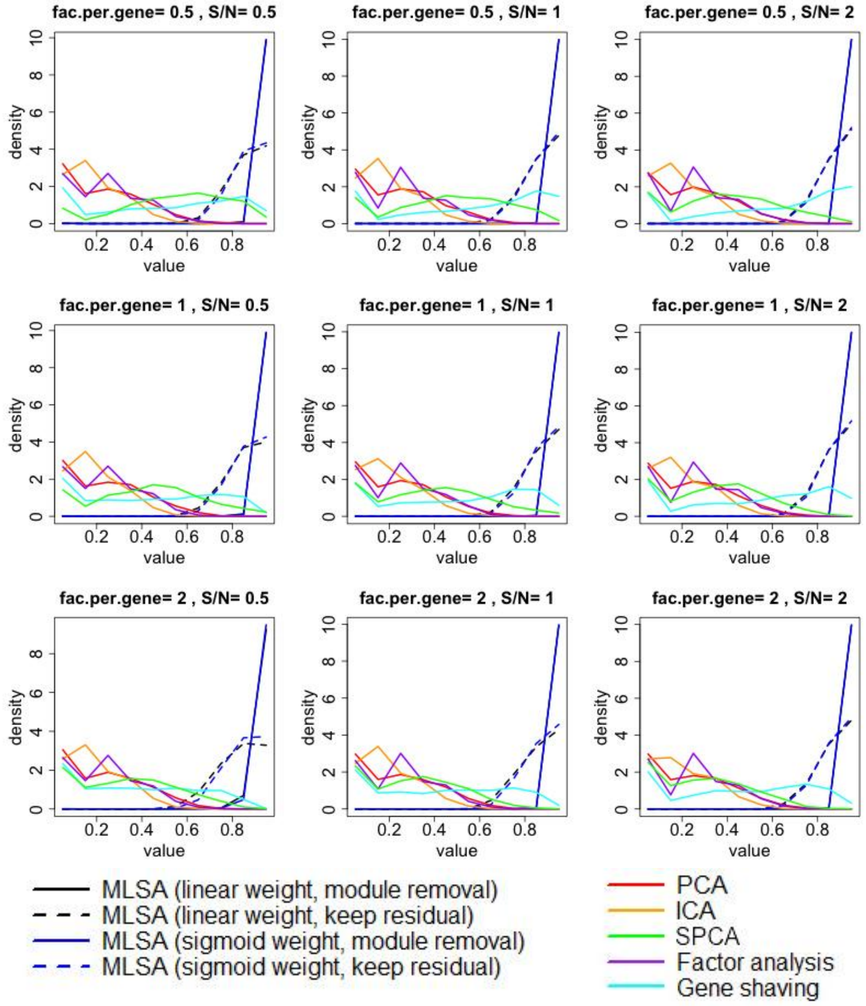

Figure S13. Simulation results from sparse global latent structure model. In every simulation, 2000 simulated genes were generated from a latent variable model with 20 latent factors. The latent factors were generated from the Gaussian distribution. Gaussian random noise was added to achieve different signal to noise ratio (columns), and different levels of sparsity were tested (rows). An additional 500 pure noise genes were generated from normal distribution. Each simulation setting was repeated 100 times. The success of latent factor recovery was evaluated by the R^2 values obtained by the regression of each latent factor against the identified factors that are most correlated with it. The relative frequencies (10 equal-sized bins between 0 and 1) of the R^2 values are plotted.

When the average number of factors per gene was 10, MLSA still maintained

considerable lead among the methods. Similar to the mixed-type factor case, all methods failed in the fully connected model (Figure S14).

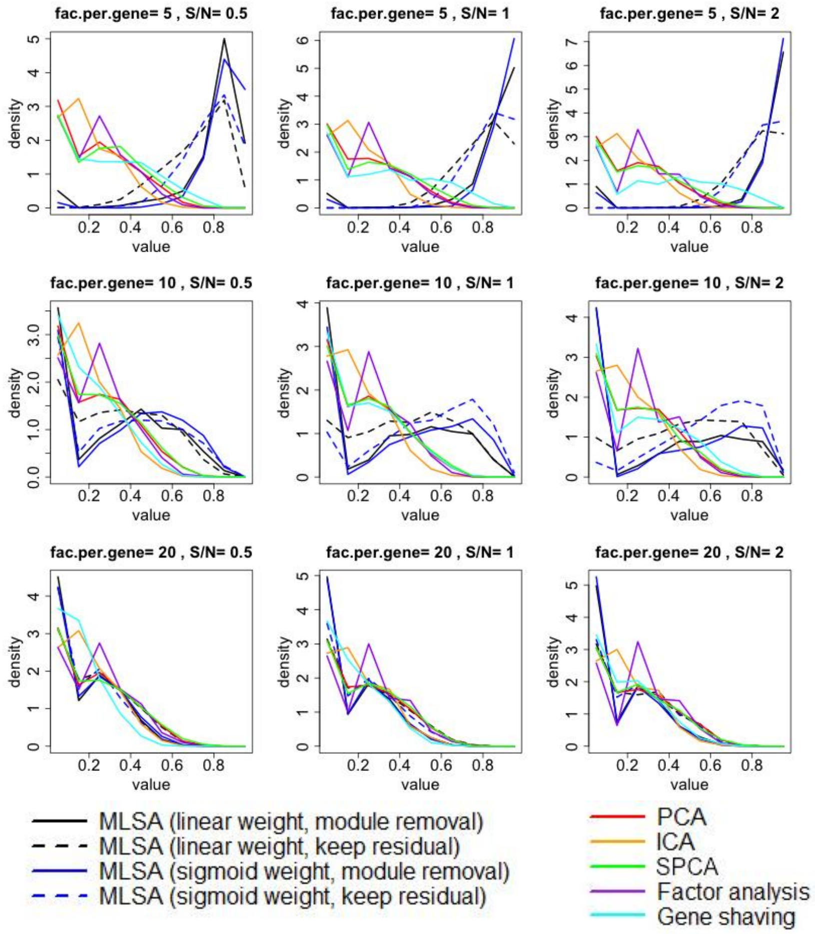

Figure S14. Simulation results from sparse global latent structure model. In every simulation, 2000 simulated genes were generated from a latent variable model with 20 latent factors. The latent factors were generated from the Gaussian distribution. Gaussian random noise was added to achieve different signal to noise ratio (columns), and different levels of sparsity were tested (rows). An additional 500 pure noise genes were generated from normal distribution. Each simulation setting was repeated 100 times. The success of latent factor recovery was evaluated by the R^2 values obtained by the regression of each latent factor against the identified factors that are most correlated with it. The relative frequencies (10 equal-sized bins between 0 and 1) of the R^2 values are plotted.
